# Ratiometric Zinc Biosensor Based on Bioluminescence Resonance Energy Transfer: Trace Metal Ion Determination with Tunable Response

**DOI:** 10.3390/ijms232314936

**Published:** 2022-11-29

**Authors:** Evgenia G. Matveeva, Andrea K. Stoddard, Hui-Hui Zeng, Graham Franke, Leslie Bourne, Carol A. Fierke, Richard B. Thompson

**Affiliations:** 1 Department of Biochemistry and Molecular Biology, University of Maryland School of Medicine, 108 N. Greene St., Baltimore, MD 21201, USA; 2 Departments of Chemistry and Biological Chemistry, University of Michigan, 930 N. University Ave., Ann Arbor, MI 48109, USA; 3 Morristown Medical Center, 100 Madison Ave., Morristown, NJ 07960, USA; 4 Department of Biochemistry, Brandeis University, 415 South St., Waltham, MA 02453, USA

**Keywords:** zinc, biosensor, bioluminescence, BRET, carbonic anhydrase, molecular imaging

## Abstract

Determination of metal ions such as zinc in solution remains an important task in analytical and biological chemistry. We describe a novel zinc ion biosensing approach using a carbonic anhydrase–*Oplophorus* luciferase fusion protein that employs bioluminescence resonance energy transfer (BRET) to transduce the level of free zinc as a ratio of emission intensities in the blue and orange portions of the spectrum. In addition to high sensitivity (below nanomolar levels) and selectivity, this approach allows both quantitative determination of “free” zinc ion (also termed “mobile” or “labile”) using bioluminescence ratios and determination of the presence of the ion above a threshold simply by the change in color of bioluminescence, without an instrument. The carbonic anhydrase metal ion sensing platform offers well-established flexibility in sensitivity, selectivity, and response kinetics. Finally, bioluminescence labeling has proven an effective approach for molecular imaging in vivo since no exciting light is required; the expressible nature of this sensor offers the prospect of imaging zinc fluxes in vivo.

## 1. Introduction

Transition metal ions, including zinc, copper and nickel, are of broad interest in part because they function both as essential nutrients and toxins in biology. In particular, zinc plays important catalytic, structural, and regulatory roles in a range of cellular processes. Although zinc is an essential nutrient, excess zinc is cytotoxic and disturbance of zinc homeostasis is associated with many health disorders. Determination of the fraction of zinc in aqueous solution which is bound by weak, readily exchangeable ligands (so-called free, labile, or mobile zinc ions) is of particular interest in biology because it is largely this fraction which is capable of binding to and thus affecting biological systems; the usually larger proportion which is tightly bound is largely unable to interact with other molecules to affect their function [1]. Since binding site occupancy is controlled by the labile zinc, knowledge of concentration of this pool remains fundamental to all aspects of zinc biology.

Over more than a century, analytical chemists have developed a series of increasingly powerful techniques to quantify metal ions, such as Zn^2+^, in aqueous solutions. Some of these methods are suitable for studying the speciation of such metal ions in solution, and in particular determining the proportion of total dissolved zinc ion bound by lower affinity, rapidly exchangeable ligands such as water or chloride ions, e.g., free zinc [2]. Methods for determining free zinc include ion-selective electrodes [3], graphite furnace atomic absorption spectroscopy [4], voltammetry [5], and numerous fluorescent indicators including small molecules [6,7,8,9,10,11] (reviewed in [12,13]) and macromolecule-based sensors using proteins or aptamers for recognition, as well as quantum dots as labels [14,15,16,17,18,19,20,21,22,23]. All of these methods rely on varyingly sophisticated instrumentation such as picoammeters, mass and optical spectrometers, fluorometers, or lasers, which in turn require electrical power, a low shock/vibration environment, non-condensing humidity, ambient air temperature near 25 °C, significant space, and/or trained personnel to operate them. Moreover, these instruments weigh tens of kg (or more), are sizable, and may be sensitive to electromagnetic interference, mechanical shock, or corrosive atmospheres. In some cases substantial preparation of samples, such as filtration or preconcentration, is necessary and the size of the sample required for trace metal determination may be significant; moreover, great care is required to avoid contamination of these samples for accurate measurements. All of these constraints make it challenging to determine free zinc at trace levels in field studies, on shipboard, and in austere sites without power or air conditioning. In most cases there must be an interposing device (often incorporating data collection and analysis in the form of a computer) that transforms the analytical signal into information describing a metal concentration that is interpretable by the operator [24]. Several groups have been developing chemical or clinical analytical approaches that require only a widely available, inexpensive device (such as a cellular telephone or “smartphone”) or no instrument at all to perform analyses [25]. Similarly, an assay for aqueous zinc that indicates the level of zinc above a low threshold without use of an instrument may be attractive for some applications. While fluorescence-based analyses of metal ions have proven sensitive, selective, and convenient for biological studies, particularly in complex matrices or in vivo, these applications are challenging in part because of the presence of background fluorescence in biological matrices and the difficulty of imaging fluorescence in many living, macroscopic organisms (reviewed in [26]) due to absorbance and scattering of biological tissues. Several workers have developed labels based on bioluminescence and chemiluminescence because these methods offer (at least in principle) very low background, and usability in vivo since no exciting light need penetrate the tissues [27]. Pioneering studies showed that zinc perturbs bioluminescent emission from firefly luciferase [28] and classic work made use of metal ion-catalyzed phthalimide chemiluminescence, but these methods have significant drawbacks for practical analyses (reviewed in [29]). Multiple investigators have developed metal ion biosensors where the presence or level of the analyte is transduced as a change in the intensity of bioluminescence of a living organism, usually a bacterium. These are attractive approaches because in many cases the organism biosynthesizes the substrate(s) for the luciferase (e.g., myristoyl aldehyde, O_2_, and FMNH_2_ for bacterial luciferase [30]) and no reagent need be added. However, the bacterium must be present and viable, the matrix must be compatible with the organism and may need to support its growth, and in many cases, it may be difficult to distinguish changes in bioluminescence due to myriad physiological factors, from those due to the analyte.

We sought to combine the high sensitivity, selectivity, and flexibility of a carbonic anhydrase (CA)-based metal ion biosensor (reviewed by [22,31,32]) with the advantages of bioluminescence to measure free zinc ion. Zinc binds with high (K_D_ = 4 picomolar) [33] affinity to the active site of human CA(II), whereas Cu(II) binds more tightly (0.1 pM) and Ni(II), Co(II), Cd(II), Fe(II) and Mn(II) all have substantially lower affinity [34,35]; the active site of CA does not bind Ca(II) or Mg(II) at concentrations as high as 10 mM or 50 mM, respectively, permitting the sensors to be used in sea water [36], as well as in bacterial and mammalian cells [20,37]. Importantly, the affinity and selectivity for zinc can be improved by subtle modification (often a single amino acid) of the CA protein [32]. Thus variants have been made with up to million-fold higher affinity for Cu(II) than Zn(II), one of which permitted measurement of free Cu(II) concentrations in living cells without Zn(II) interference [38]. Similarly, the metal ion binding kinetics may also be improved by nonrandom mutagenesis of CA, such that the association rate constant for zinc binding can be enhanced 800-fold over the wild-type with only a 10-fold loss in affinity [39]. By intelligent design of the sensor fluorophores, the zinc concentration can be transduced as a change in fluorescence emission or excitation wavelength ratios, polarization (anisotropy) or lifetime, all of which offer well-established advantages over simple intensity measurements in fluorescence sensing [40,41,42,43]. Therefore, we chose to adapt our fluorescence-based zinc biosensor to bioluminescence to take advantage of these same benefits.

## 2. Results

### 2.1. Principle of the Sensor

**Figure 1:**
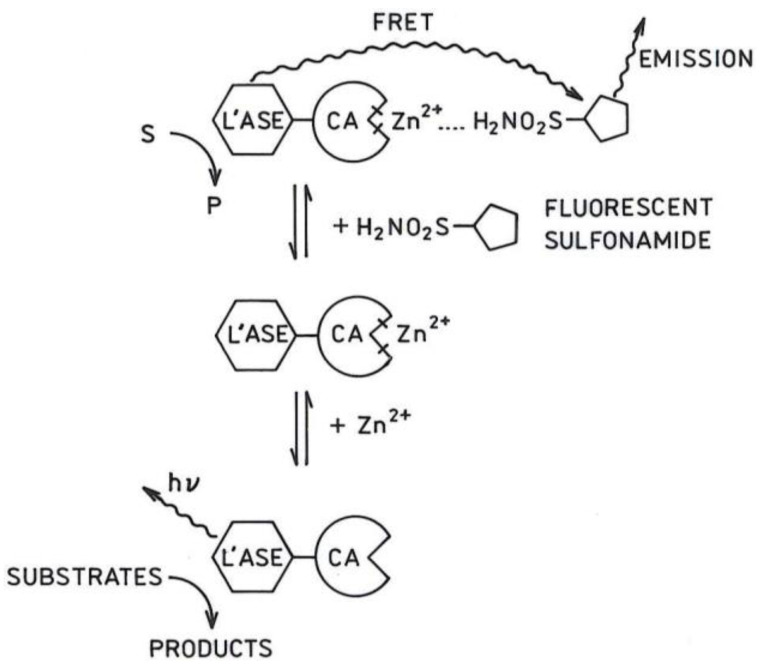
Principle of the sensor. In the figure, “L’ASE” indicates schematically the luciferase portion of the fusion protein, “CA” the carbonic anhydrase portion of the fusion protein, and “FRET”, Förster resonance energy transfer.

The sensor is based on the zinc-dependent ratiometric bioluminescence emission of a carbonic anhydrase II- luciferase fusion protein (CA-LUC); the principle is illustrated in Figure 1.

Briefly, in the absence of zinc ion but with the addition of the luciferase substrate(s), one observes the typical bioluminescence emission of the luciferase from the CA-luciferase fusion. Addition of a fluorescent sulfonamide derivative chosen first to have an overlap of its absorbance with the luciferase emission, and secondly to bind to the CA active site with micromolar or higher affinity when zinc is present, in the absence of zinc does not bind to apo-CA-luciferase, and does not alter the typical luciferase emission. However, when zinc binds to the apo-CA, the affinity of the sulfonamide to CA increases significantly, so the sulfonamide binds to form a CA-Zn-sulfonamide complex [44]. Once bound, the sulfonamide serves as a Förster resonance energy transfer (FRET; in this context sometimes called BRET for bioluminescence) acceptor for the bioluminescent excited state of the oxidized luciferin due to its close proximity, and emits at longer wavelengths characteristic of the fluorophore, with a corresponding decline in the bioluminescence of the luciferase. We tested fusion proteins employing either firefly luciferase from *Photinus pyralis* which utilizes luciferin and ATP as substrates, and emits at 560 nm [28], or luciferase modified from the naturally occurring enzyme in a deep sea shrimp, *Oplophorus gracilirostris*, which uses a coelenterazine derivative and oxygen as substrates, and emits around 460 nm [45]. Ultimately, the *Oplophorus* luciferase-CA construct gave a substantial ratiometric response to varying free zinc concentrations; results of those experiments are shown below.

### 2.2. Experimental Results

In our initial experiments using wild type firefly luciferase-CA fusion proteins the energy transfer to the bound sulfonamide was modest despite excellent overlap between the luciferase emission (λmax = 560 nm) and the Rhodamine absorption (λmax = 560 nm) leading to a Forster distance of R_0_ = 54.8 Å (results not shown). The reason for this modest signal is difficult to isolate without knowing the structure of the fusion protein. Possibilities include that the distance between the bound luciferin donor and the sulfonamide acceptor is large or that the fusion with CA destabilizes the structure of luciferase such that energy transfer or quantum yield of the donor is decreased. In view of these issues, we used a recently developed variant of the luciferase from *Oplophorus gracilirostris*, a deep sea shrimp. This enzyme is small (19 kDa vs. 62 kDa for firefly luciferase), consists of a single domain, and is relatively stable [45]. Furthermore, it catalyzes a bioluminescent reaction similar to that found in coelenterates, such as *Renilla*, using coelenterazine and oxygen as substrates and producing oxidized coelenterazine, light, and CO_2_ as products. The variant luciferase and gene are available commercially as NanoLuc (Promega), together with a modified imidapyrazinone substrate, furimazine. We fused the genes for NanoLuc to the N- or C-termini of CA to form His_6_-NanoLuc-CA and His_6_-CA-NanoLuc, respectively, expressed these proteins in *E. coli* and purified them by metal affinity chromatography. The CA-NanoLuc and CA-firefly luciferase (luciferase fused at C-terminus of the CA) constructs gave weaker energy transfer in response to variations in the zinc concentration than the NanoLuc-CA construct [46], and the former were not pursued further.

**Figure 2:**
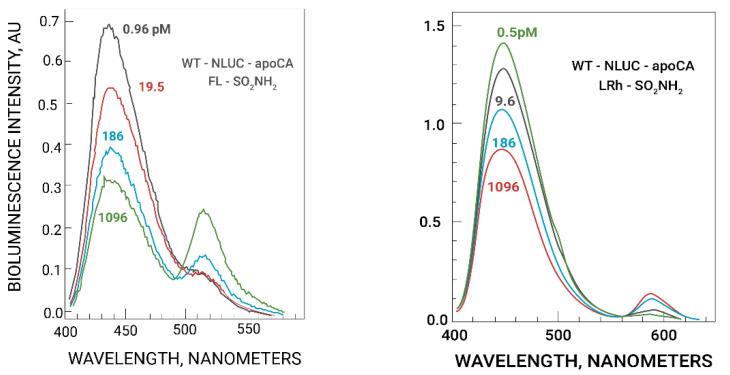
Zinc-dependent emission spectra of wt-NLUC-apoCA with fluorescein sulfonamide (**left panel**) or Lissamine Rhodamine sulfonamide (**right panel**). Reading from the top at 450 nm the free zinc concentrations are (**left panel**) 0.96 pM, 19.5 pM, 186 pM, and 1.096 nM; and (**right panel**) 0.5 pM, 9.6 pM, 186 pM, and 1.096 nM.

In a typical assay 1.0 µM NanoLuc-apoCA construct and 2.1 µM fluorescent sulfonamide were incubated with MOPS NTA zinc buffers, then 15 µM furimazine was added to start the reaction. Following brief mixing bioluminescence spectra were obtained; typical spectra are depicted in Figure 2. In the HEPES buffer the bioluminescence decays more rapidly (t_½_ ~ 8 min) compared with the proprietary buffer (t_½_ ~ 2 h) [45]. We found that although the overall intensity declined by up to 40% over five minutes, during that time the ratio of peak intensities changed only 4% (results not shown). Thus, rapid mixing of the reagents is unnecessary for accurate measurements.

The fluorescein sulfonamide gives significantly greater emission than the Rhodamine sulfonamide (Figure 2). This is expected because the overlap of the fluorescein sulfonamide absorbance peak at 495 nm with the NanoLuc luciferase emission peak at 450 nm is significantly greater than that of the Lissamine Rhodamine sulfonamide peak at 568 nm, and they have comparable peak extinction coefficients (77,000 and 88,000 M^−1^ cm^−1^, respectively). Using Förster’s theory and the known spectra and refractive indices and estimated orientation factor κ^2^ = 2/3 we calculated Förster distances (R_0_, the distance where donor:acceptor energy transfer should be 50% efficient) [47] of 48.1 and 40.2 Ångstroms for the fluorescein and rhodamine sulfonamides, respectively. In view of the known sizes of the NanoLuc luciferase (PDB 5IBO) and human carbonic anhydrase II (PDB 1CA2) (approximately 20 and 26 Å in diameter, respectively) we expect that energy transfer will be highly efficient even though the structure of the fusion protein and the relative orientation of the donor and acceptor are unknown. This is evident in the approximately 70% decline of the bioluminescence (donor) emission as the zinc site (and sulfonamide site) become saturated (see Figure 2). There may be differences as well in the relative orientations and quantum yields of the two fluorophores that contribute to the apparent difference in energy transfer efficiency.

In the absence of addition of either the fluorescent sulfonamide or zinc no increase in emission corresponding to the sulfonamide wavelengths was observed; similarly, no emission was obtained in the presence of non-luciferase-fused carbonic anhydrase (results not shown). Furthermore, emission at the sulfonamide wavelengths can be abolished by addition of an excess of a much tighter binding (K_D_ ≈ 40 nM vs. ≈1 µM) sulfonamide, acetazolamide (Diamox), that competes successfully with the fluorescein or rhodamine sulfonamide for binding to CA, indicating that the energy transfer occurs with specifically bound sulfonamide and not to the molecule free in solution.

Since the fractional occupancy of the zinc binding site in the carbonic anhydrase active site is a simple function of the free zinc concentration when the zinc is buffered [48,49], the concentration of free zinc is proportional to the ratio of emission from the sulfonamide energy transfer acceptor to that from the luciferase donor. This can be seen in Figure 2 (left) for NanoLuc-apoCA with fluorescein sulfonamide, and in Figure 2 (right) for NanoLuc-apoCA with Lissamine Rhodamine sulfonamide.

**Figure 3:**
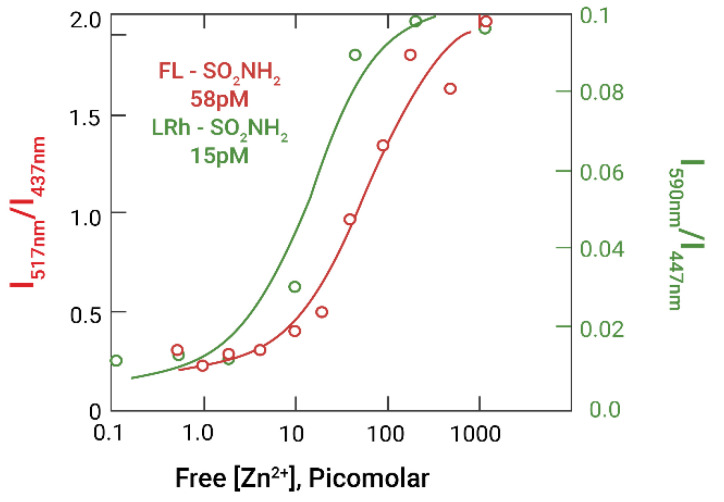
Zinc-dependent bioluminescence intensity ratios and best fit binding isotherms of NanoLuc-apoCA with Fluorescein sulfonamide (I_517 nm_/I_437 nm_, ○–○) (red) and Lissamine Rhodamine sulfonamide (I_590 nm_/I_447 nm_, ○–○) (green).

In the presence of zinc buffers, the ratios of fluorescein and rhodamine sulfonamide emission intensities to luciferase emission intensity as a function of free zinc concentration yield the binding isotherms shown in Figure 3 rather than a linear dependence on zinc concentration; fitting the data to a single binding site yields similar K_D_ values of 58 ± 15 and 15 ± 6 pM, respectively.

The data in Figure 3 make it apparent that the system is capable of assaying free zinc ion under these conditions below the nanomolar range, with a detection limit around 5 picomolar. We note that the zinc ion affinity of wild type human CA II alone is approximately 4 picomolar under similar conditions [35] which is clearly tighter than the 15–58 pM affinity of the NanoLuc-CA fusion protein described here. We have made several examples of chemically modified or gene-fused wild type CA molecules which exhibit decreased zinc affinities (up to 2 decades weaker in K_D_) than the wild type protein, as well as many variants with mutations of residues in or near the active site with both higher and lower zinc affinities (reviewed in [31,32]). We propose that the short linker between NanoLuc and CA may slightly alter the structure of the zinc binding site and decrease the binding affinity compared to the native CA; efforts are ongoing to examine the origin of the reduced affinity. We hypothesize that the slight difference in zinc affinity exhibited using the two sulfonamides may be attributed to the formation of the CA-Zn-sulfonamide complex being a coupled equilibrium and the sulfonamides having slightly different affinities for holoCA themselves; experiments are under way to verify this. Based on the demonstrated changes in our carbonic anhydrase fluorescence-based zinc biosensors, we expect that the sensitivity, selectivity, and kinetics of the bioluminescence-based sensors can be similarly modified by mutation of the carbonic anhydrase moiety.

**Figure 4:**
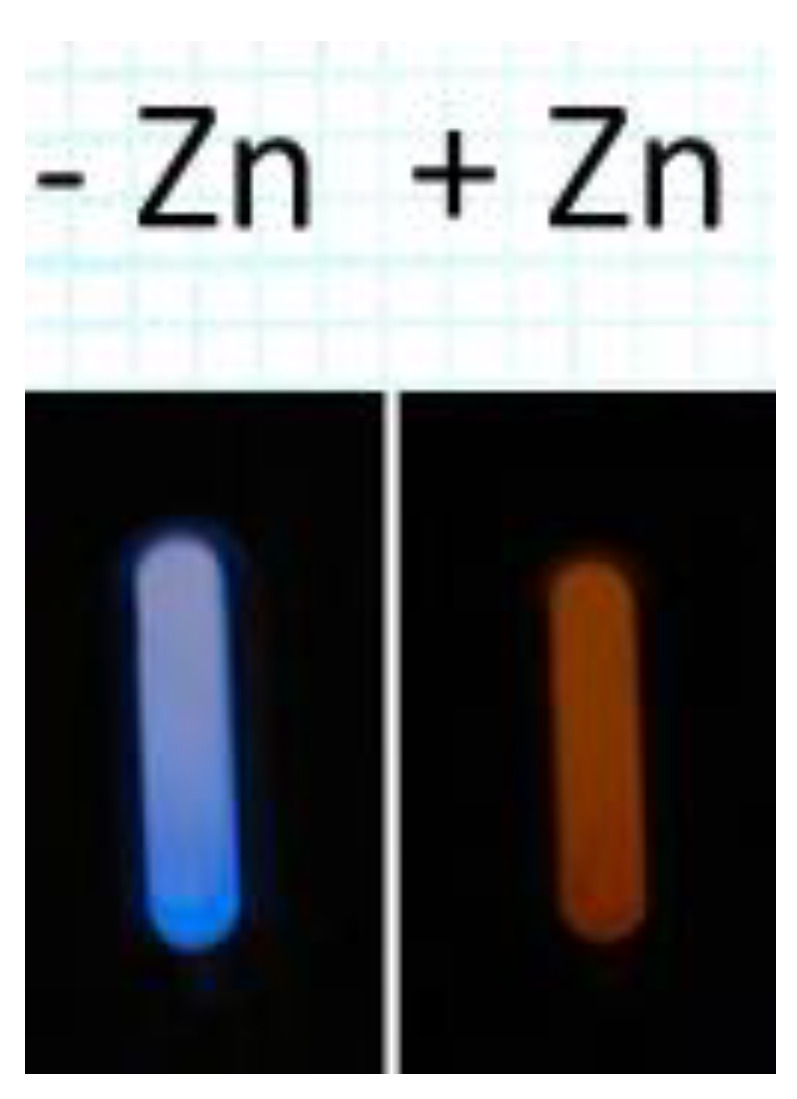
Photographs of furimazine-induced bioluminescence of apoNanoLuc-CA (−Zn^2+^, left) and holoNanoLuc-CA (+Zn^2+^, right), both in the presence of Lissamine Rhodamine sulfonamide taken with Nikon D100 SLR with 60 mm AF Micro-Nikkor 60 mm f/2.8, 2 s exposure, with no other illumination; Schott KV550 filter on right.

Finally, we note that, as described in the Introduction most analyses of aqueous zinc require an instrument, a skilled operator, and substantial resources. While many simple analytical tools that are usable without an instrument (such as test strips) exist, most rely on color changes and consequently have modest (micromolar) sensitivity. By comparison, this approach offers sub-nanomolar sensitivity of zinc detection in a device that gives a visible change in color of the bioluminescence (Figure 4); one could imagine constructing the assay for a particular threshold value of zinc, which if exceeded would be apparent to the operator unambiguously.

## 3. Discussion

This bioluminescence resonance energy transfer-based Zn^2+^ sensing approach has several important advantages for quantitative determinations: first, unlike many bioluminescent and chemiluminescent assays it is ratiometric. A frequent issue with bioluminescence-based assays is the kinetics of bioluminescence emission, which can be roughly divided into “flash” emission and “glow” emission for fast and slow emission, respectively. Substantial effort has been devoted to understanding and controlling bioluminescence emission kinetics in assays because of the respective advantages and disadvantages of slow and fast kinetics. For instance, rapid kinetics typically provide more sensitivity because the brief burst of light is easier to detect above background, but at the same time requires a luminometer with rapid, highly reproducible mixing to provide accurate results. Conversely, a very slow bioluminescent emission rate does not require rapid mixing but offers lower sensitivity since the light flux is spread out in time. In a fashion precisely analogous to the classic T-format polarization fluorometer of Weber [50] or the wavelength ratiometric fluorescent calcium indicators of Grynkiewicz et al. [40], the ratio of direct bioluminescent emission to FRET acceptor emission changes only slightly for the first few minutes of bioluminescence emission, since the rate of energy transfer is very fast (>10^8^ s^−1^) and the association rate constant of the sulfonamide is typically of the order of 10^5^ M^−1^ s^−1^ [51], indicating that at these concentrations sulfonamide binding equilibrates in milliseconds.

An important attribute of this approach is that bioluminescence labels have been demonstrated as very useful for in vivo molecular imaging of, for example, tumors in experimental animals [27]. Due to the extensive absorption and scattering of tissue at visible and near-IR wavelengths [52], there is substantial depth-dependent attenuation of the excitation for fluorescent labels, even in small animals. By comparison, the essentially chemical nature of the bioluminescence excitation means that no exciting light need penetrate the tissue, only the emission. Thus from the standpoint of in vivo imaging and quantifying zinc fluxes, this approach has advantages for studying zinc biology in the brain, pancreas, intestine, prostate, and other tissues. There remain significant challenges in introducing both the Nanoluc-CA and the fluorescent sulfonamide into the desired tissue in an experimental animal, but clearly many groups have succeeded in expressing and imaging luciferase constructs of many proteins (such as antibodies) in living animal models (reviewed in [27]) as well as imaging fluorophores in in vivo models and humans [53,54]. We have had success expressing CA fusion proteins in multiple tissue culture cell types, introducing labeled CA into cells using TAT tags or microinjection, and using CA on the distal end of fiber optics to measure intracerebral free zinc [20,37,55,56]. Similarly, the decades-long clinical employment of aryl sulfonamide CA inhibitors suggests that their pharmacology is well understood and can be optimized for a given application [57]; we note that because there is no background emission from the unbound fluorescent sulfonamide, it can in principle be given systemically. We also note that we have demonstrated fluorescent sulfonamide inhibitors with emission in the infrared [58,59]; we view the flexibility of using different fluorescent sulfonamides as an advantage of this approach. Experiments are underway to test the sensor in a suitable animal model.

We note that the Merkx group in their pioneering study [60] demonstrated a BRET-based zinc biosensor based on a large conformational change induced by zinc between a luciferase domain and a fluorescent protein domain in a fusion protein Very recently, they have reported improvements to this system with improved affinity and a larger change in intensity ratio [61]. While their approach has the advantage of simplicity in that it does not require a separate aryl sulfonamide, we believe the use of the aryl sulfonamide offers certain advantages: in particular, the emission at the acceptor wavelength can be minimized and thus accurately determined by removal of the sulfonamide (or competing it off with a higher affinity sulfonamide), such that no energy transfer can occur. Additionally, we and others have described a very diverse group of sulfonamides that emit at wavelengths into the infrared [59], which may be particularly useful for tissue measurements in vivo.

## 4. Materials and Methods

Materials: Firefly luciferin and ATP were purchased from Sigma, furimazine was from Promega, Chelex-100 resin was from BioRad; buffers were formulated using ACS reagent grade salts and were stripped of divalent cations by passage through a Chelex-100 column. Genes for firefly luciferase or NanoLuc (Promega) were fused to the N- or C-terminus of variants of human carbonic anhydrase II with an alanyl-alanyl linker between the two genes and a Tev protease-cleavable 6-His tag on the C-terminus. These genes were subcloned into the pET expression vector pET20b+ and the protein was expressed in *E. coli* BL21(DE3) after induction with IPTG, purified by anion exchange and nickel affinity chromatography, and the 6-His tag removed by treatment with Tev protease, all essentially as described previously [20]. The NLuc-CA construct gave a substantially larger signal than the CA-NLuc construct or either of the firefly luciferase constructs; the latter three were not pursued further. Zinc was removed from the protein by treatment with 2,6-dipicolinic acid at pH 7.0 in Tris buffer, essentially as previously described [62]. The Lissamine Rhodamine sulfonamide and fluorescein sulfonamide were synthesized by reaction of 4-(2-aminoethyl) benzenesulfonamide (Aldrich) with Lissamine Rhodamine sulfonyl chloride or fluorescein isothiocyanate, respectively (both Life Technologies) in pH 8.0 buffer essentially as previously described [58] and purified by silica gel column chromatography.

Methods: Bioluminescence spectra were obtained on a Spectronics AB-2 spectrophotofluorometer without excitation using slit widths of 4 or 8 nm and PMT levels of 600–800 volts. Bioluminescence assays were performed in 20 mM HEPES or MOPS pH 7.3 buffers rather than the proprietary reaction buffer supplied by Promega which contains ingredients incompatible with accurate measurement of free zinc concentrations. Metal ion buffers were formulated with 2 mM NTA, 20 mM HEPES or MOPS, and micromolar total concentrations of ZnCl_2_ using MINEQL software (Environmental Research Software, Hallowell, Maine) to provide free zinc ion concentrations ranging from 0.5 pM to 10 nM, essentially as previously described [48].

## 5. Conclusions

We describe here a bioluminescence-based free zinc ion biosensor employing the carbonic anhydrase recognition moiety that offers high and tunable sensitivity, ratiometric response, and simple implementation.

## 6. Patents

R.B. Thompson, E.G. Matveeva, C. A. Fierke, L. Bourne, G. Franke, “Bioluminescent metal Ion Assay” U.S. Patent No. 9,193,990 B2 (Appl. No. 14/218,085) 24 November 2015.

## Data Availability

Numerical data are available upon request from the corresponding author.
